# Risk factors for venous thromboembolism following spinal surgery: A meta-analysis

**DOI:** 10.1097/MD.0000000000020954

**Published:** 2020-07-17

**Authors:** Lu Zhang, Hongxin Cao, Yunzhen Chen, Guangjun Jiao

**Affiliations:** a Department of Orthopedics, Qilu Hospital of Shandong University and Spine and Spinal Cord Disease Research Center, Shandong University; b Department of Medical Oncology, Qilu Hospital of Shandong University, Jinan, Shandong, China.

**Keywords:** deep vein thrombosis, meta-analysis, risk factors, spine surgery, venous thromboembolism

## Abstract

**Background:**

To investigate the risk factors for postoperative venous thromboembolism (VTE) in patients undergoing spinal surgery.

**Methods:**

Literature published in PubMed, Embase, the Cochrane Library, and Web of Science was systematically reviewed to assess risk factors for VTE following spinal surgery. The data analysis was conducted with STATA 12.0. Data were pooled using fixed-effects or random-effects models according to the heterogeneity among the included studies.

**Results:**

Twenty-six studies involving 3,216,187 patients were included in this meta-analysis, and the total incidence of VTE after spinal surgery was 0.35% (0.15–29.38%). The pooled analysis suggested that the incidence of VTE after spinal surgery was higher in such aspects as increasing age (weighted mean difference [WMD] 0.55 years, 95% confidence interval [CI] 0.33–0.78, *P* < .001), female sex (odds ratio [OR] 1.12, 95% CI 1.01–1.25; *P* = .034), diabetes (OR 1.34, 95% CI 1.29–1.44; *P* < .001), chronic kidney disease (OR = 8.31, 95% CI 1.98–34.93; *P* = .004), nonambulatory preoperative activity status (OR 3.67, 95% CI 2.75–4.83; *P* < .001), D-dimer level (WMD 1.023, 95% CI 0.162–1.884; *P* = .02), long duration of operation (WMD 0.73, 95% CI 0.21–1.24; *P* = .006), spine fusion (OR 1.54, 95% CI 1.31–1.82; *P* < .001), and blood transfusion (OR 2.31, 95% CI 1.73–3.07; *P* < .001), and the differences were statistically significant. However, there were no significant differences in body mass index, obesity, hypertension, coronary heart disease, spondylolisthesis, intraoperative blood loss, surgical procedures (anterior lumbar interbody fusion vs posterior intervertebral fusion /translaminar lumbar interbody fusion), or surgical site (lumbar vs thoracic) (all *P* > .05).

**Conclusion:**

Based on our meta-analysis, we identified several important factors that increased the risk of VTE after spinal surgery. We hope our study provides assistance to spine surgeons so that they can adequately analyze and assess risk factors in patients and then develop preventive measures to reduce the incidence of VTE.

## Abstract 2

Supplemental Digital Content is available in the text

## 1 Introduction

Venous thromboembolism (VTE), including pulmonary embolism (PE), and deep venous thrombosis (DVT), are well-known and feared complications of major orthopedic surgery.^[1,2]^ VTE may result in severe morbidity with poor quality of life and even sudden death, increase hospital costs and bring a heavy burden to patients and families. Protocols for the use of drug prophylaxis are well established for some surgeries, most notably hip and knee arthroplasty.^[3]^ The recommendations for chemoprophylaxis in spinal surgery are less clear, and clear evidence-based guidelines are lacking.^[4]^ Given the relative paucity of consensus concerning the efficacy and safety of chemoprophylaxis in spinal surgery, management varies widely and tends to be carried out according to surgeons’ personal experiences.^[5]^ Therefore, it is very necessary for spine surgeons to be aware of the incidence of VTE and its risk factors.

Spinal surgery is unique because its bleeding complications can lead to an epidural hematoma, which is a devastating but comparatively rare complication with an approximated incidence of <1%.^[6–8]^ Therefore, spine surgeons should be more cautious when making decisions regarding anticoagulant therapy. They have to face the dilemma of how to balance the risk of morbidity and mortality from VTE and the risk of permanent neurological injury from epidural hematomas. Numerous studies have focused on the risk factors for VTE in patients undergoing spine surgery.^[9–18]^ The incidence varies on account of these relatively small studies, which are conducted in heterogeneous populations using various detection methods. Collectively, these studies indicate that patients with an older age undergoing long periods of bedrest for paralysis and pain are reported to have a higher incidence of VTE than other patients.^[9,10,12–14]^ While the incidence of VTE after spine surgery is influenced by other factors, such as D-dimer level, duration of operation, intraoperative blood loss, and surgical procedures, the resulting incidence of VTE after spine surgery is not inconsistent or ever contradictory.

Therefore, we carried out this study to systematically review and meta-analyze the existing literature to more precisely describe the incidence of VTEs and the risk factors associated with VTEs for patients who underwent spine surgery; we aimed to summarize the evidence-based proof for this issue, with the goal of supporting surgeons to make wise clinical decisions.

## 2 Materials and methods

### 2.1 Ethics statement

There was no need to seek informed consent from patients as this meta-analysis was based on published data and there was no potential harm to patients; this was approved by Ethics Committee of Qilu Hospital of Shandong University.

### 2.2 Search strategy

A PRISMA (preferred reporting items for systematic reviews and meta-analyses)-compliant search was performed in the PubMed, Embase, Cochrane Library, and Web of Science databases by using combinations of the following keywords: venous thromboembolic, pulmonary embolism, deep venous thrombosis, spine surgery, and spinal surgery. References cited in the relevant articles were also reviewed. All studies were carefully reviewed to identify repeated data.

### 2.3 Inclusion and exclusion criteria

Studies were included if they conformed to the following criteria: original articles; randomized or nonrandomized controlled studies; studies on spinal surgery; studies on VTE after spine surgery; and studies on patients with spine degeneration diseases. Studies that were duplicate reports of an earlier trial, reviews, letters, and case reports were excluded.

### 2.4 Selection of studies

Two investigators independently reviewed all subjects, abstracts, and the full text of the selected literature. Then, the eligible studies were selected according to the inclusion criteria. Any controversy between the investigators was resolved by discussion and consensus. When consensus could not be reached, a 3rd author was consulted to resolve the disagreement.

### 2.5 Data extraction and quality assessment

Two independent authors extracted the data from the eligible studies, discussed discrepancies, and reached a consensus for all items. The essential information extracted from all primary studies included the titles, author names, year of publication, study location, study design, sample size, number of patients with VTE, demographic characteristics (age, sex, body mass index [BMI], obesity, hypertension [HT], heart disease [HD], diabetes, spondylolisthesis, chronic kidney disease [CKD]), the level of D-dimer, preoperative walking disability, spine fusion vs nonfusion, anterior lumbar interbody fusion (ALIF) vs posterior intervertebral fusion (PLIF)/translaminar lumbar interbody fusion (TLIF), surgical duration, blood loss, and blood transfusion. We used the modified Newcastle–Ottawa scale (NOS) to evaluate the quality of the studies. This method consists of 3 items: selection, comparability, and outcomes. Scores ranged from 0 to 9, and higher scores indicated better quality. Any study was considered to be of high quality if the NOS score was >5 points.^[19]^

### 2.6 Statistical analysis

The STATA 12.0 (Stata Corporation, College Station, TX) was used to analyze the data. Dichotomous variables are expressed as odds ratios (ORs) with 95% confidence intervals (CIs), and continuous variables are expressed as weighted mean differences (WMDs) with 95% CIs. A probability of *P* < .05 was considered statistically significant. Heterogeneity was tested using *I*^2^ statistic.^[20]^ If *I*^2^ >50%, heterogeneity was implied, and a random-effects model (DerSimonian–Laird method)^[21]^ was used; if *I*^2^ < 50%, a fixed-effects model (Mantel–Haenszel method)^[21]^ was used.^[21]^ When significant heterogeneity was identified, a sensitivity analysis was performed to explore the potential source of heterogeneity. Publication bias was evaluated with Begg^[22]^ and Egger^[23]^ test.

## 3 Results

### 3.1 Search results

**Figure 1:**
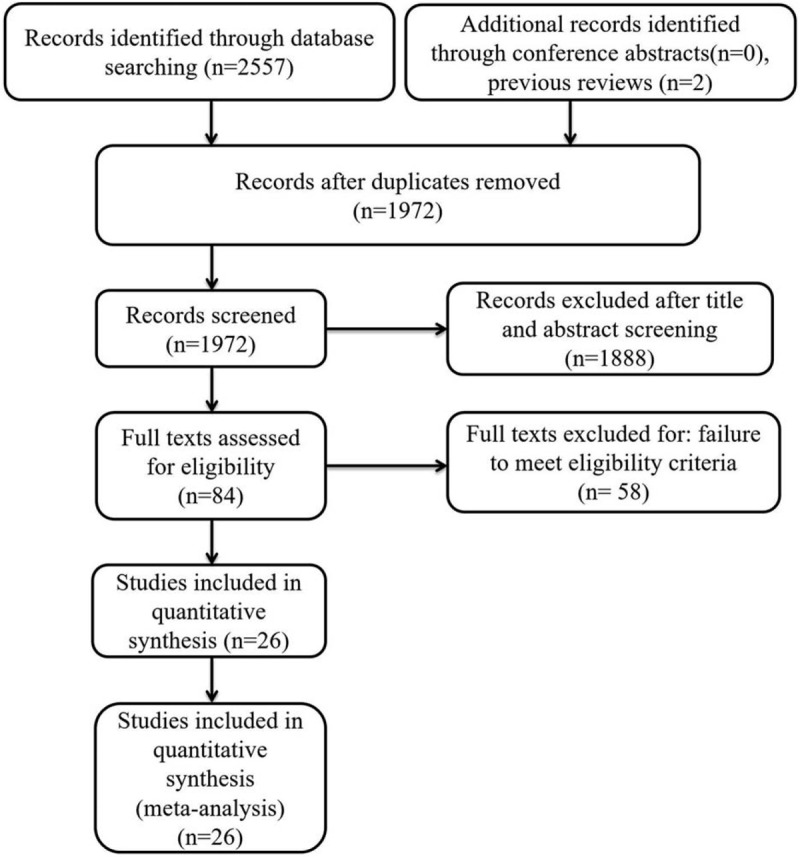
The flow diagram of the selection process for relative studies.

The article screening and selection process for inclusion in this study is shown in Figure 1. A total of 2559 studies were initially identified. Of these, 587 were excluded for duplicate records, and 1888 were excluded after the review of the title/abstract. Then, 84 studies remained for the full-text review. Among these, 58 were excluded for failure to meet the eligibility criteria. Finally, 26 studies met the inclusion criteria and were included in our meta-analysis, and their characteristics are presented in Table 1. One of these 26 studies^[9–18,24–39]^ was designed as a prospective study, and the other 25 are retrospective studies. The total sample size was 3,216,187, of which 1196 were complicated with VTE, and the total incidence of VTE after spinal surgery was 0.35% (the incidence of VTE in the original studies was 0.15–29.38%). The incidence of VTE was 8.43% in Asian patients and 0.33% in occidental patients, with a statistically significant difference (*P* < .0001).

**Table 1:**
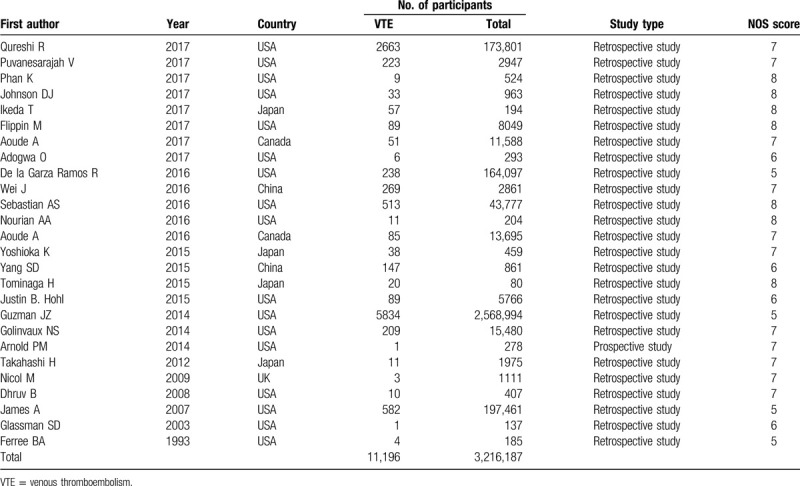
Baseline characteristics of patients in trials included.

### 3.2 Quality assessment

Assessment of the study-specific quality scores from the NOS is shown in Table 1. The median score of the included studies was 7, with a range from 5 to 8, which indicated that these studies were of high quality.

### 3.3 Risk factors

#### 3.3.1 Increased age

**Figure 2:**
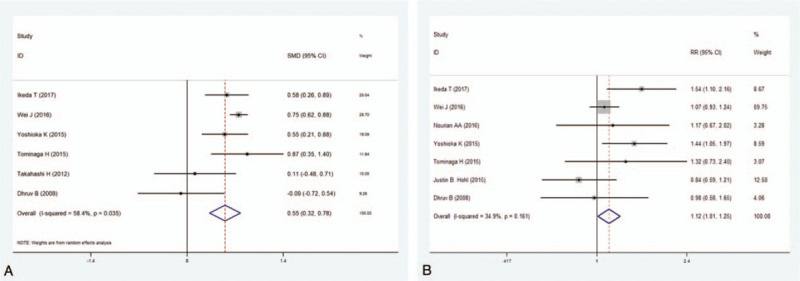
(A) Forest plot showing relationship between increased age and incidence of venous thromboembolism (VTE) after spine surgery. (B) Forest plot showing the relationship between female sex and incidence of VTE after spine surgery. CI = confidence interval, OR = odds ratio, SMD = standardized mean difference.

The most important risk factors for VTE are presented in Table 2. Six studies^[9,10,12,14,16,37]^ including 6218 patients investigated the relationship between increased age and the incidence of VTE. The pooled results suggested that spinal surgery patients with VTE were older than those without VTE (WMD 0.55 years, 95% CI 0.33–0.78, *P* < .001; *I*^2^ = 58.4%, *P* = .035; Fig. 2A).

**Table 2:**
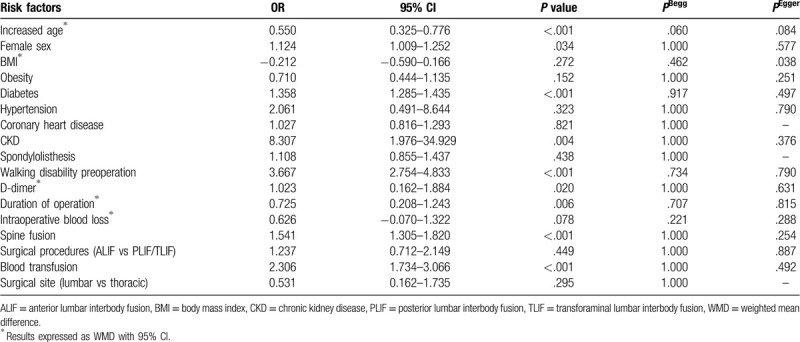
Pooled estimates of OR (WMD)^∗^ obtained from meta-analysis of risk factors of venous thromboembolism following spine surgery.

#### 3.3.2 Female sex

Seven studies^[9,10,12,14,15,31,37]^ including 9973 patients assessed the relationship between sex and the incidence of VTE after spinal surgery. The pooled estimate showed that females had a significantly higher incidence of VTE than males (OR 1.12, 95% CI 1.01–1.25; *P* = .034; Fig. 2B). There was no significant heterogeneity among the studies (*I*^2^ = 34.9%, *P* = .161).

#### 3.3.3 Body mass index

Five studies^[9,10,12,14,16]^ including 5572 patients reported the relationship between BMI and the incidence of VTE. Pooled estimates indicated that patients with high BMI values had a similar risk of VTE compared to those with normal BMI (WMD −0.21 kg/m^2^, 95% CI −0.59 to 0.17; *P* = .272; Supplemental Fig. 1). There was significant heterogeneity (*I*^2^ = 85.5%, *P* < .001); thus, we conducted subgroup analyses. When we excluded a Chinese study,^[10]^ the pooled data for the remaining Japanese studies^[9,12,14,16]^ did not change substantially (WMD −0.35 kg/m^2^, 95% CI −0.74 to 0.04; *P* = .077), but there was still heterogeneity present (*I*^2^ = 70.9%, *P* = .016).

#### 3.3.4 Obesity

Three studies^[26,28,31]^ including 8777 patients reported the relationship between obesity and the incidence of VTE. The pooled results suggested that obesity patients had a similar incidence of VTE compared to those of normal weight (OR 0.71, 95% CI 0.44–1.13; *P* = .152; Supplemental Fig. 2). There was no significant heterogeneity among the studies (*I*^2^ = 0.0%, *P* = .600).

#### 3.3.5 Diabetes

**Figure 3:**
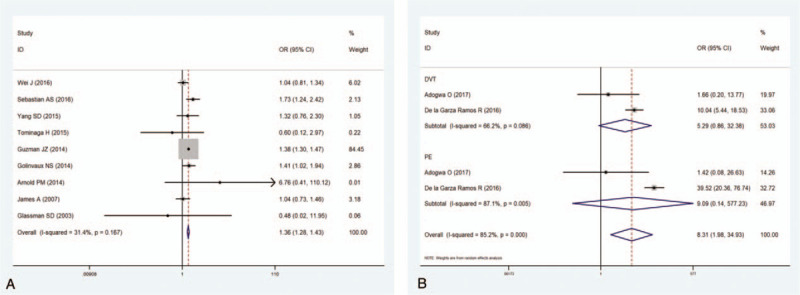
(A) Forest plot showing the relationship between diabetes and incidence of venous thromboembolism (VTE) after spine surgery. (B) Forest plot showing the relationship between chronic kidney disease and incidence of VTE after spine surgery. CI = confidence interval, OR = odds ratio.

Nine studies^[10,11,13,14,34–36,38,39]^ including 2,866,773 patients assessed the relationship between diabetes and the incidence of VTE. The pooled data suggested that diabetes patients had a 1.34-fold increased risk of VTE compared to those without diabetes (OR 1.34, 95% CI 1.29–1.44; *P* < .001; Fig. 3A). There was no significant heterogeneity among the studies (*I*^2^ = 31.4%, *P* = .167).

#### 3.3.6 Hypertension

Four studies^[10,11,13,14]^ including 47,172 patients investigated the relationship between HT and the incidence of VTE. The pooled estimate suggested that HT patients had a similar incidence of VTE compared to that of normal patients (OR 2.06, 95% CI 0.49–8.64; *P* = 0.323; Supplemental Fig. 3). There was significant heterogeneity among the studies (*I*^2^ = 98.5%, *P* < .001). Thus, we conducted sensitivity analyses. When we excluded a study with a relatively small sample (n = 80), the pooled data for the remaining studies changed dramatically (OR 2.40, 95% CI 2.09–2.76; *P* < .001), but there was still heterogeneity present (*I*^2^ = 99.0%, *P* < .001).

#### 3.3.7 Coronary heart disease

Two studies^[10,13]^ including 3725 patients reported a relationship between coronary HD and the incidence of VTE. The pooled results indicated that patients with coronary HD had a similar incidence of VTE compared to normal patients (OR 1.03, 95% CI 0.82–1.29; *P* = .821; Supplemental Fig. 4). There was no significant heterogeneity among the studies (*I*^2^ = 0.0%, *P* = .948).

#### 3.3.8 Chronic kidney disease

Two studies^[30,32]^ including 328,240 patients reported a relationship between CKD and the incidence of VTE. The results showed that patients with CKD had an 8.31-fold increased risk of VTE compared to those without CKD (OR = 8.31, 95% CI 1.98–34.93; *P* = .004). Heterogeneity was significant (*I*^2^ = 85.2%, *P* < .001). Thus, a subgroup analysis was performed (Fig. 3B).

#### 3.3.9 Spondylolisthesis

Two studies^[10,31]^ including 3068 patients investigated the relationship between spondylolisthesis and the incidence of VTE. The results suggested that patients with spondylolisthesis had a similar risk of VTE compared to those without spondylolisthesis (OR = 1.11, 95% CI 0.86–1.44; *P* = .438; Supplemental Fig. 5). There was no significant heterogeneity among the studies (*I*^2^ = 0.0%, *P* = .517).

#### 3.3.10 Nonambulatory preoperative activity status

**Figure 4:**
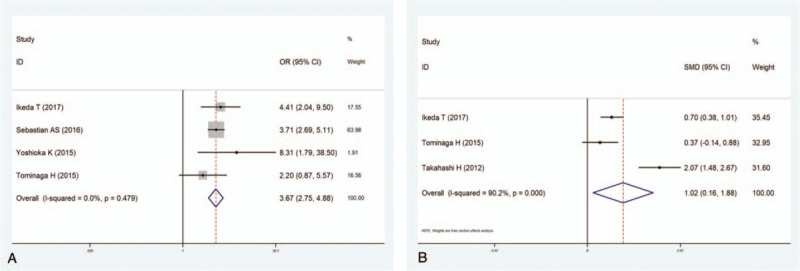
(A) Forest plot showing the relationship between preoperative activity nonambulatory status and incidence of venous thromboembolism (VTE) after spine surgery. (B) Forest plot showing the relationship between high level of serum D-dimer and incidence of VTE after spine surgery. CI = confidence interval, OR = odds ratio, SMD = standardized mean difference.

Four studies^[9,11,12,14]^ including 44,510 patients investigated the relationship between a nonambulatory preoperative activity status and the incidence of VTE. The pooled results suggested that a preoperative walking disability notably increased the incidence of VTE (OR 3.67, 95% CI 2.75–4.83; *P* < .001; Fig. 4A). There was no significant heterogeneity among the studies (*I*^2^ = 0.0%, *P* = .479).

#### 3.3.11 D-dimer level

Three studies^[9,14,16]^ including 2249 patients investigated the relationship between a high level of serum D-dimer and the incidence of VTE. The results revealed that a high level of D-dimer prominently increased the incidence of VTE (WMD 1.023, 95% CI 0.162–1.884; *P* = .02; Fig. 4B). The heterogeneity among the studies was significant (*I*^2^ = 90.2%, *P* < .001).

#### 3.3.12 Duration of operation

**Figure 5:**
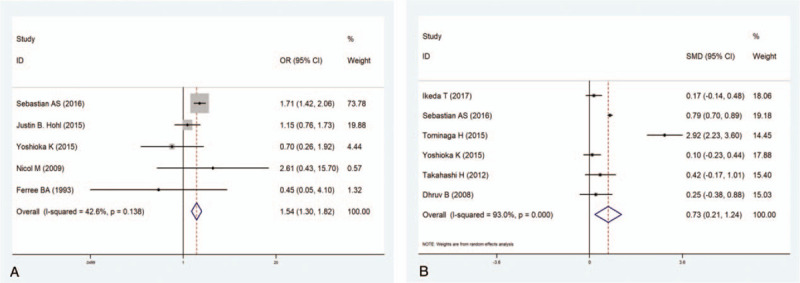
(A) Forest plot showing the relationship between duration of operation and incidence of venous thromboembolism (VTE) after spine surgery. (B) Forest plot showing the relationship between spine fusion surgery and incidence of VTE after spine surgery. CI = confidence interval, OR = odds ratio, SMD = standardized mean difference.

Six studies^[9,11,12,14,16,37]^ including 46,891 patients reported the relationship between the duration of the operation and the incidence of VTE. The results revealed that long surgical times dramatically increased the incidence of VTE (WMD 0.73, 95% CI 0.21–1.24; *P* = .006; Fig. 5A). The heterogeneity among the studies was significant (*I*^2^ = 93.0%, *P* < .001).

#### 3.3.13 Intraoperative blood loss

Five studies^[9,12,14,16,37]^ including 3114 patients observed a relationship between intraoperative blood loss and the incidence of VTE. The results indicated that intraoperative blood loss did not affect the incidence of VTE (WMD 0.63, 95% CI −0.07 to 1.32; *P* = .08; heterogeneity: *I*^2^ = 91.4%, *P* < .001, Supplemental Fig. 6).

#### 3.3.14 Spine fusion

Five studies^[11,12,15,17,18]^ including 49,278 patients researched the relationship between spine fusion and the incidence of VTE. The pooled results showed that spine fusion significantly increased the incidence of VTE (OR 1.54, 95% CI 1.31–1.82; *P* < .001; Fig. 5B). No significant heterogeneity was observed (*I*^2^ = 42.6%, *P* = .138).

#### 3.3.15 ALIF vs PLIF/TLIF

Three studies^[11,24,37]^ including 179,844 patients investigated the difference in VTE risk between ALIF patients and PLIF/TLIF patients. The results suggested that ALIF patients had a similar risk of VTE compared to PLIF/TLIF patients (OR 1.24, 95% CI 0.71–2.15; *P* = .45; heterogeneity: *I*^2^ = 68.4%, *P* = .04, Supplemental Fig. 7).

#### 3.3.16 Blood transfusion

**Figure 6:**
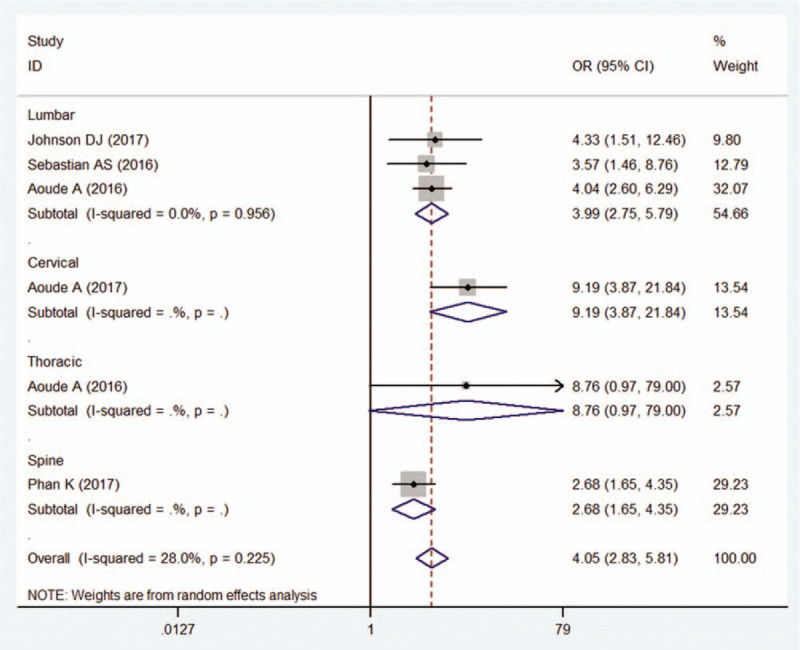
Forest plot showing relationship between blood transfusion and incidence of venous thromboembolism after spine surgery. Subgroup analysis according to the surgery site was performed. CI = confidence interval, OR = odds ratio.

Five studies^[11,25,27,29,33]^ including 72,493 patients assessed the relationship between a blood transfusion and the incidence of VTE. As 1 study^[33]^ included the patients undergoing lumbar and thoracic fusion surgery, another study^[29]^ included patients undergoing cervical fusion surgery, subgroup analysis was performed according to the surgery site. The pooled data revealed that a blood transfusion remarkably increased the incidence of VTE (OR 4.050, 95% CI 2.825–5.805; *P* < .001). No significant heterogeneity was observed (*I*^2^ = 28.0%, *P* = .225). Thus, a subgroup analysis was performed according to the surgical site (Fig. 6).

#### 3.3.17 Surgical site

Two studies^[14,33]^ including 13,761 patients investigated the difference in VTE risk between patients undergoing lumbar fusion surgery and patients undergoing thoracic fusion surgery. The pooled data indicated that patients undergoing lumbar fusion surgery had a similar risk of VTE compared to patients undergoing thoracic fusion surgery (OR 0.531, 95% CI 0.162–1.735; *P* = .295; heterogeneity: *I*^2^ = 23.8%, *P* = .252, Supplemental Fig. 8).

#### 3.3.18 Publication bias

The assessment of publication bias using Begg and Egger tests showed that there was no potential publication bias for any of the included studies (Table 2).

## 4 Discussion

The incidence of VTE in the field of orthopedics varies with surgical site or surgical procedure. Compared with the incidence of VTE after lower limb surgery, such as hip or knee arthroplasty, the incidence of VTE after spinal surgery is relatively low. Nonetheless, if not promptly treated, thromboembolic complications, such as lower extremity DVT, PE, myocardial infarction, and cerebral infarction after spinal operation, could lead to severe malfunction of the extremities, heart, and brain, and even death. VTE after spine surgery has drawn increasing attention from researchers; however, the incidence of and risk factors for VTE following spine surgery remain unclear. This meta-analysis summarized and analyzed the risk factors for VTE after spine surgery. The difference in the incidence of VTE between Asians and occidentals was also investigated. The results indicated that Asian ethnicity, older age, being female, preoperative walking disability, high levels of serum D-dimer, longer surgical times, spine fusion surgery, and blood transfusion, combined with diabetes or CKD, were risk factors for VTE after spine surgery.

A total of 3,216,187 patients from 26 studies were included in this study, including 11,196 patients with VTE after spine surgery. The overall incidence of VTE was 0.35%, which was much lower than in a previous meta-analysis.^[40]^ In addition, it was found that the incidence of VTE in Asian patients was significantly higher than that in occidental patients, which indicated that Asian patients were more likely to suffer from VTE after spine surgery; the reason for this remains to be investigated by further research. Our study indicated that elderly patients were more likely to develop VTE, which was in line with previous studies.^[9,10,12]^ In a retrospective study of 2861 patients that investigated risk factors for DVT in patients undergoing posterior lumbar interbody fusion, the authors suggested that age was an independent risk factor for DVT.^[10]^ The mean age in the DVT and non-DVT groups was 61.3 and 52.6 years (*P* = .002), respectively,^[10]^ suggesting that elderly age increased the risk of DVT. In some of the studies included in this meta-analysis, although the mean age of the VTE group was older than that of the non-VTE group, no significant difference was shown. Tominaga et al^[14]^ conducted a retrospective clinical study and performed a logistic regression analysis of 80 patients who had undergone spine surgery. Among these patients, 20 experienced postoperative VTE, and 60 had no VTE. The median ages for the patients with VTE and those without VTE were 75.0 and 70.5 years, respectively, and they were not statistically significant (*P* = .075).^[14]^ Likewise, Takahashi et al^[16]^ undertook a review of a case series to identify the incidence of VTE after spinal surgery. The results showed that the mean age did not differ significantly between the PE and non-PE groups (67.1 vs 65.5 years old, respectively). We think that the respectively small sample size and selection bias are influencing factors of the results. To obtain more objective conclusions, we pooled all the data from the included studies together in a meta-analysis to weaken the bias of a single study. As is commonly known, aging patients tend to have a high incidence of diabetes and cardiovascular and cerebrovascular diseases and have a long postoperative bedrest period after surgery. Therefore, it is reasonable that elder patients are at higher risk for VTE. Increasing age is one of the risk categories in the Autar DVT scale.^[3]^ Attention must be paid to elderly patients after spinal surgery, and steps must be taken to prevent the incidence of VTE.

Consistent with prior studies, female sex was significantly associated with VTE after spine surgery.^[9,12]^ Yoshioka et al^[12]^ and Ikeda et al^[9]^ found that female sex was one of the risk factors in multivariate analyses. However, in the other 5 studies,^[10,14,15,31,37]^ a negative relationship was found between female sex and VTE. The pooled data indicated that females had a significantly higher incidence of VTE than males, and there was no significant heterogeneity among the studies. We consider that female patients recover more slowly and stay in bed longer after spine surgery than male patients, thereby increasing the risk of VTE.

Diabetes was another risk factor for VTE following spine surgery, and this result was comparable to previous studies.^[11,34,35]^ Guzman et al^[34]^ performed a retrospective database analysis. They enrolled a total of 403,629 controlled diabetic patients, 19,421 uncontrolled diabetic patients, and 2,145,944 nondiabetic patients who underwent degenerative lumbar spine surgery. They found that relative to nondiabetic patients, uncontrolled diabetic patients had significantly increased odds of having DVT (*P* < .001); moreover, controlled diabetic patients also had an increased risk of DVT when compared with nondiabetic patients (*P* < .001).^[34]^ However, some other studies had the opposite idea that a history of diabetes would not increase the incidence of VTE.^[10,14,38]^ Our results suggested that a history of diabetes markedly increased the incidence of VTE. It is known that hyperglycemia and endocrine metabolic disturbances may lead to hormone imbalances in the whole body.^[40,41]^ In addition, patients who have diabetes are more prone to vascular stenosis, which affects hemodynamics, thereby increasing the risk of VTE.

There are few studies on CKD and VTE. De la Garza Ramos et al^[32]^ analyzed 164,097 patients who underwent treatment for degenerative spine disease. Among these, 1047 had CKD, and 270 had ESRD (end-stage renal disease). After multiple logistic regression analyses, the authors found that patients with CKD and ESRD were dramatically more likely to experience DVT and PE when compared with patients without kidney disease. Another study had the opposite idea, that is, that the incidence of PE after lumbar decompression and fusion was similar between the CKD group and the no-CKD group.^[30]^ Our meta-analysis revealed that a history of CKD was a significant risk factor for VTE in patients who had undergone spinal surgery. This may be related to the imbalance of the internal environment and anemia in patients with CKD. Due to the significant heterogeneity (*I*^2^ = 85.2%, *P* < .001), we performed a subgroup analysis. However, the subgroup analysis data suggested that the incidences of both PE and DVT were similar between CKD patients and non-CKD patients. Therefore, further studies are needed to determine whether CKD is a risk factor for VTE.

In the present study, we found that blood transfusion was a remarkable risk factor for VTE in patients who had undergone spinal surgery. These results were in line with previous studies.^[11,27,29,33]^ Three studies^[11,27,33]^ observed a relationship between postoperative transfusion and the incidence of VTE in patients who had undergone lumbar spinal surgery. Aoude et al^[33]^ retrospectively analyzed the American College of Surgeons National Surgical Quality Improvement Program (ACS-NSQIP) database to identify patients who underwent lumbar or thoracic fusion. They enrolled 13,695 patients, of whom 13,170 had lumbar fusion and 525 had thoracic fusion. In 2016, they reported that patients receiving transfusions who underwent lumbar fusion were more likely to develop VTE, which was in line with the other 2 studies,^[11,27]^ while there was no increased risk of VTE in patients receiving transfusions who underwent thoracic fusion. Again, Aoude et al^[29]^ identified 11,588 patients who had cervical fusion from the ACS-NSQIP database. Their results suggested that all transfused patients had an increased risk of VTE. It was assumed that the postoperative transfusion increased the viscosity of the blood, which may affect hemodynamics and the blood coagulation mechanism and that this may be correlated with an increased risk of VTE postoperatively.

### 4.1 Limitations

Some limitations of this study should be recognized. First, the sample size was huge, so the chance of internal errors, significant heterogeneity among the studies and samples, and bias was also high. Consequently, in some results, observable heterogeneity was identified among the included studies. Despite sensitivity analyses and subgroup analyses being performed to detect potential sources of heterogeneity, no valuable information was observed. Second, clinical heterogeneity might be caused by the different indications for surgery, different postoperative bedrest times and the postoperative functional rehabilitation exercises used at different treatment centers. Moreover, all of the studies were performed with a retrospective design, and their results might be biased by inherent shortcomings. This may have had a potential impact on our pooled estimates.

## 5 Conclusion

Our study indicates that Asian ethnicity, advanced age, female sex, diabetes, CKD, high levels of D-dimer, preoperative walking disability, long duration of operation, spine fusion, and blood transfusion are independent risk factors for VTE following spine surgery, whereas BMI, obesity, HT, coronary HD, spondylolisthesis, intraoperative blood loss surgical procedures, and surgical site are not. Knowing these risk factors, surgeons can fully analyze and assess risk factors in patients and then formulate preventive measures to reduce the incidence of VTE.

## Author contributions

**Data curation:** Yunzhen Chen.

**Formal analysis:** Hongxin Cao.

**Investigation:** Hongxin Cao.

**Software:** Hongxin Cao.

**Supervision:** Yunzhen Chen.

**Writing – original draft:** Lu Zhang.

**Writing – review & editing:** Jiao Guangjun.

## Supplementary Material

Supplemental Digital Content
