# Supplementary material for: Risk factors for venous thromboembolism following spinal surgery: A meta-analysis
Source: Medicine (Baltimore). 2020 Jul 17;99(29):e20954. doi: 10.1097/MD.0000000000020954 (PMC7373537; doi:10.1097/MD.0000000000020954)
Supplement: Supplemental Digital Content [file medi-99-e20954-s001.docx]

**Supplement Figures**

**
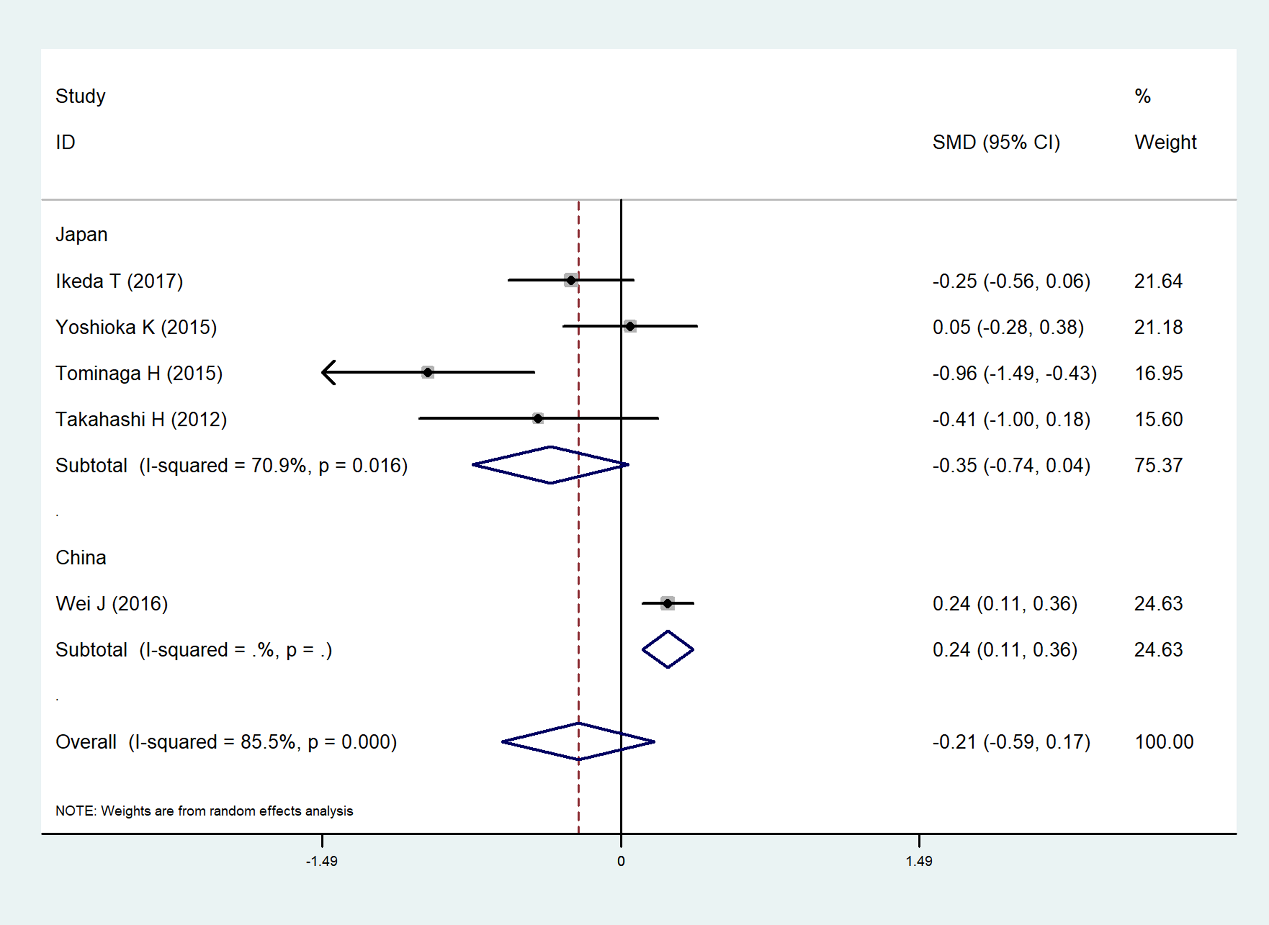
**

Supplement Figure 1. Forest plot showing relationship between BMI and incidence of VTE after spine surgery.


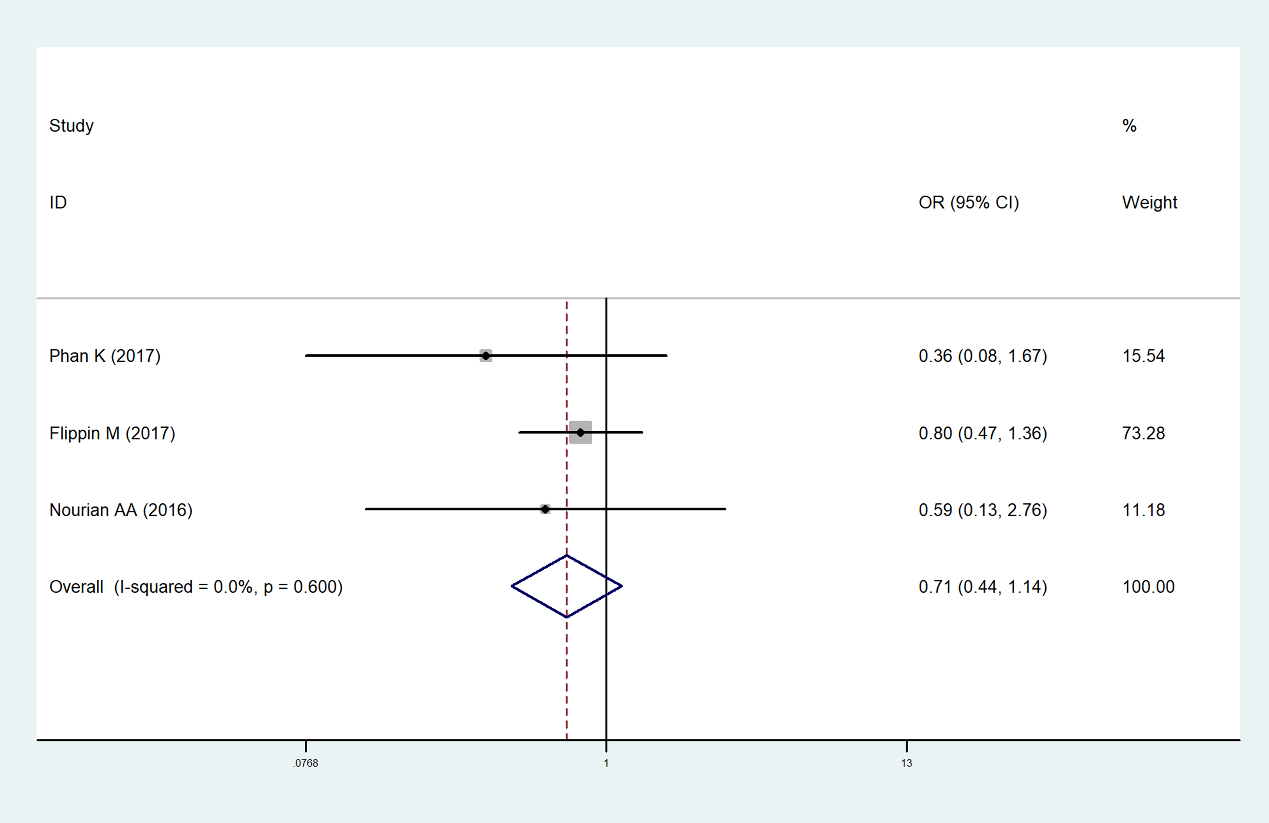


Supplement Figure 2. Forest plot showing relationship between obesity and incidence of VTE after spine surgery.


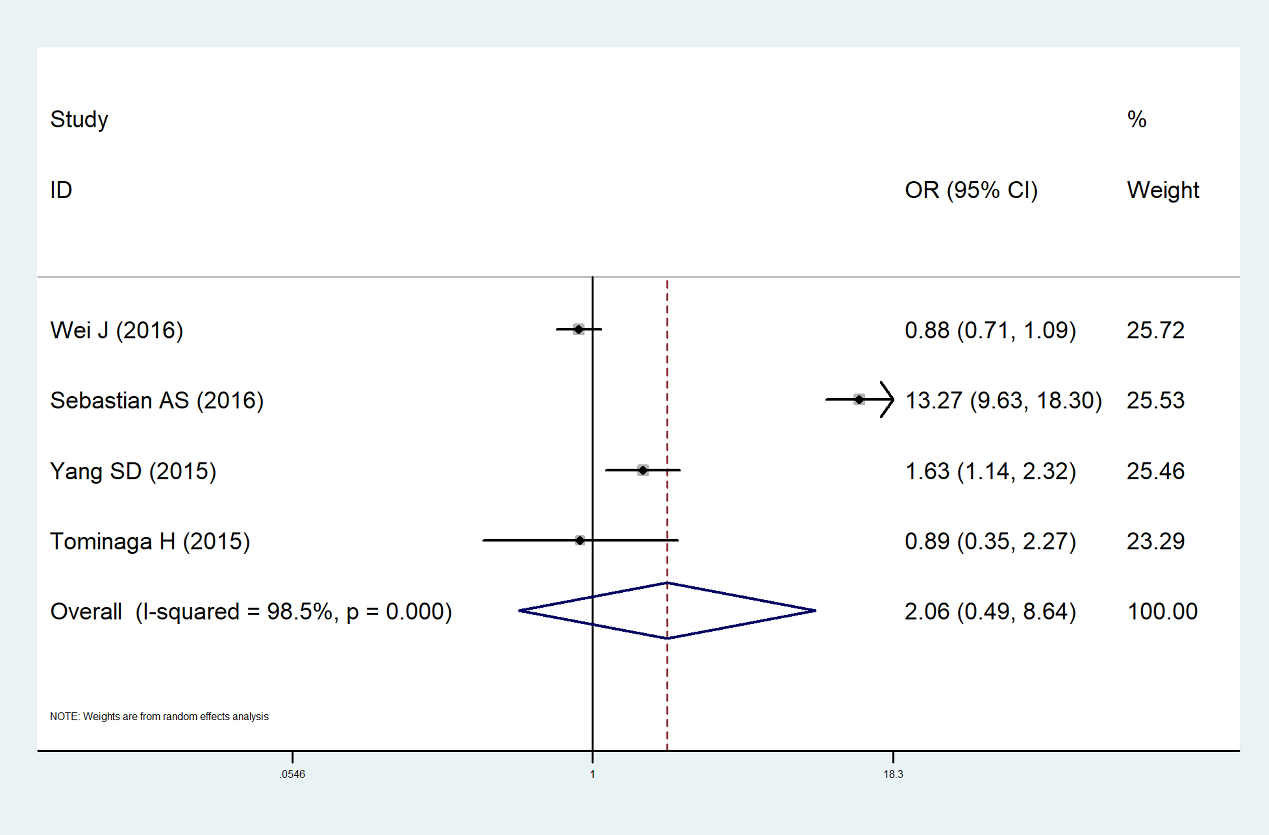


Supplement Figure 3. Forest plot showing relationship between hypertension and incidence of VTE after spine surgery.


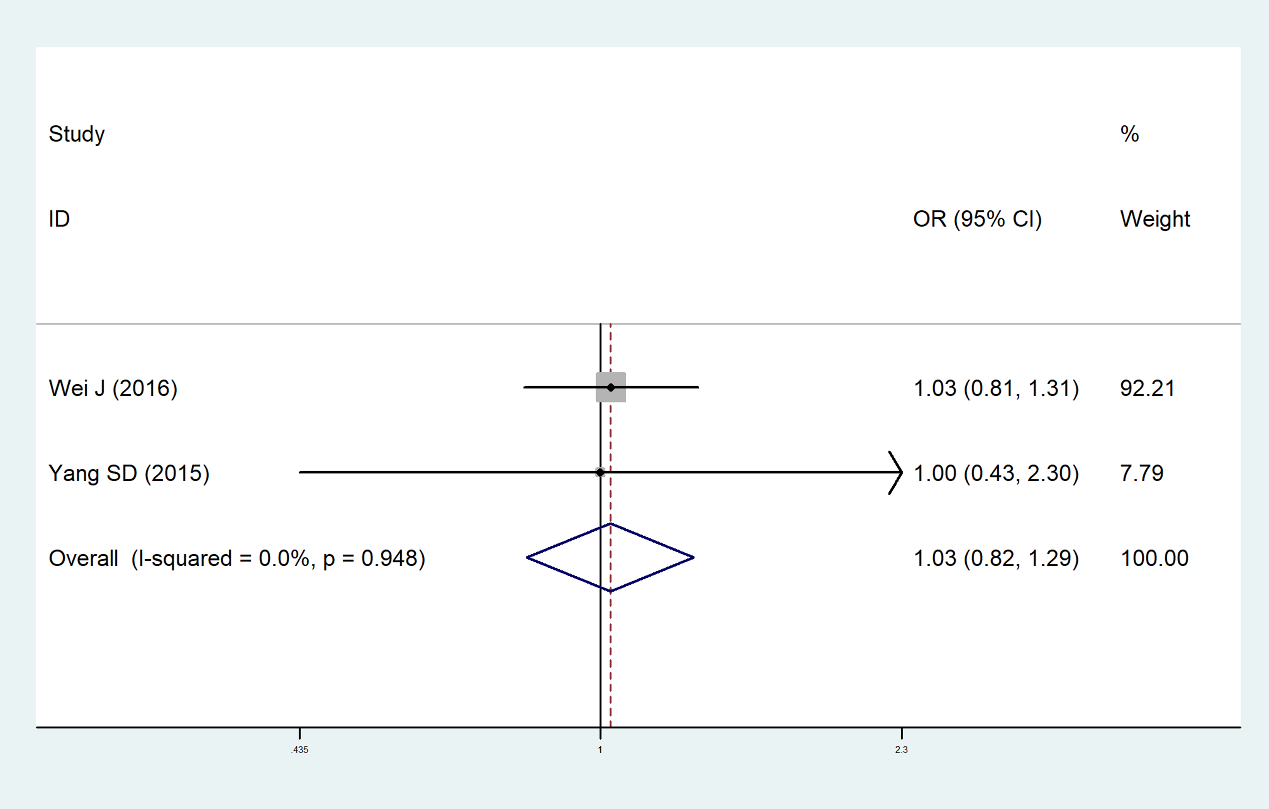


Supplement Figure 4. Forest plot showing relationship between coronary heart disease and incidence of VTE after spine surgery.


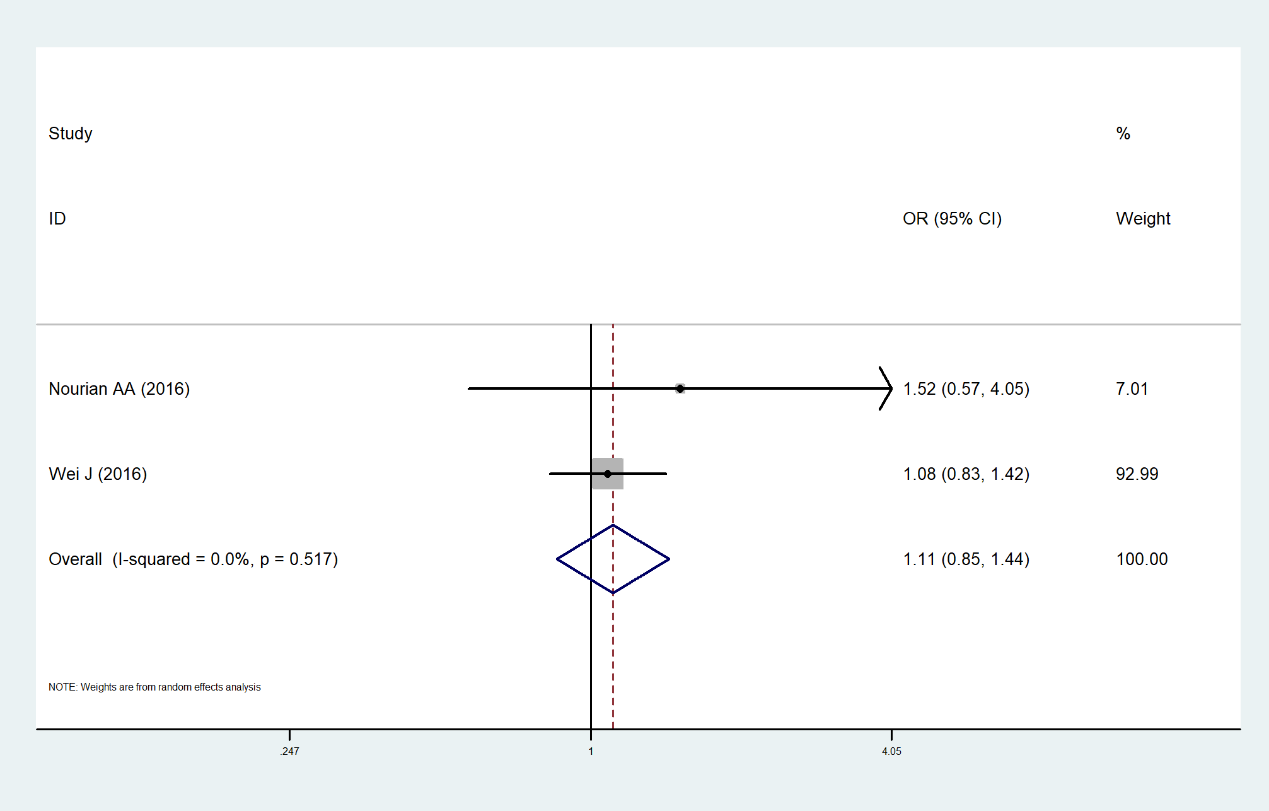


Supplement Figure 5. Forest plot showing relationship between spondylolisthesis and incidence of VTE after spine surgery.


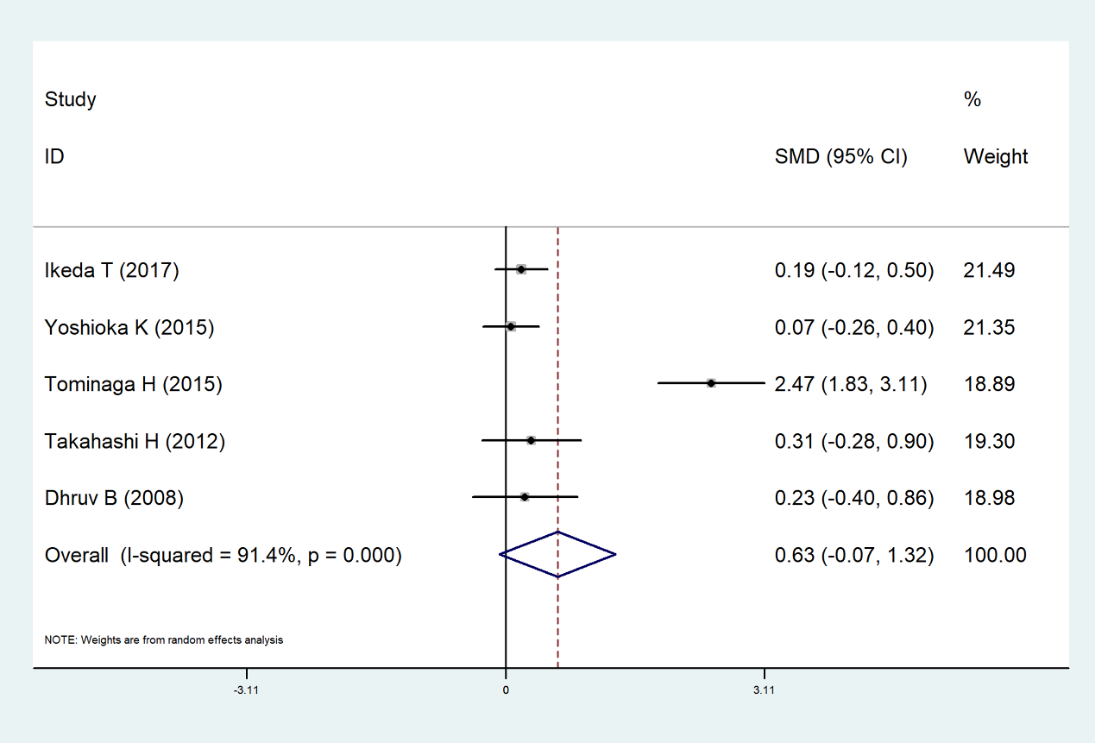


Supplement Figure 6. Forest plot showing relationship between intraoperative blood loss and incidence of VTE after spine surgery.


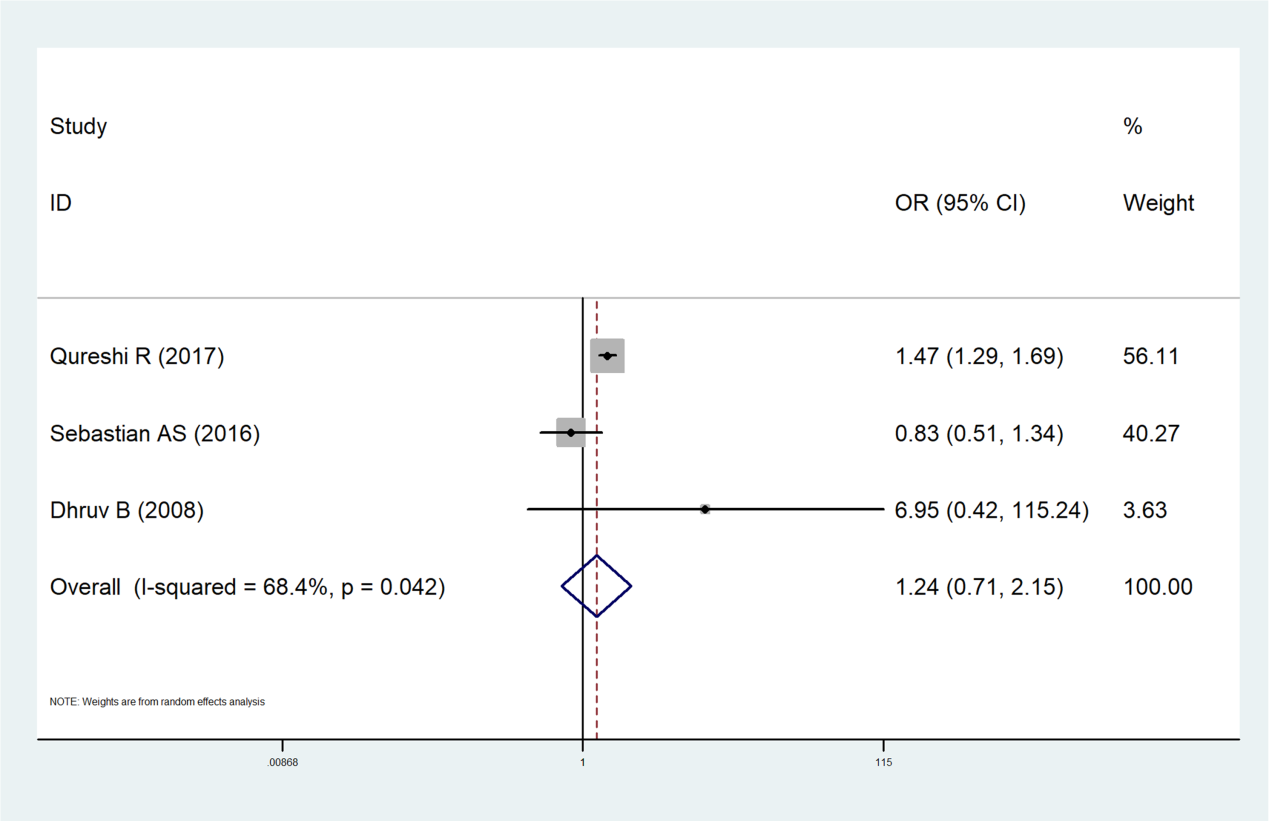


Supplement Figure 7. Forest plot showing relationship between surgical procedures (ALIF vs PLIF/TLIF) and incidence of VTE after spine surgery.


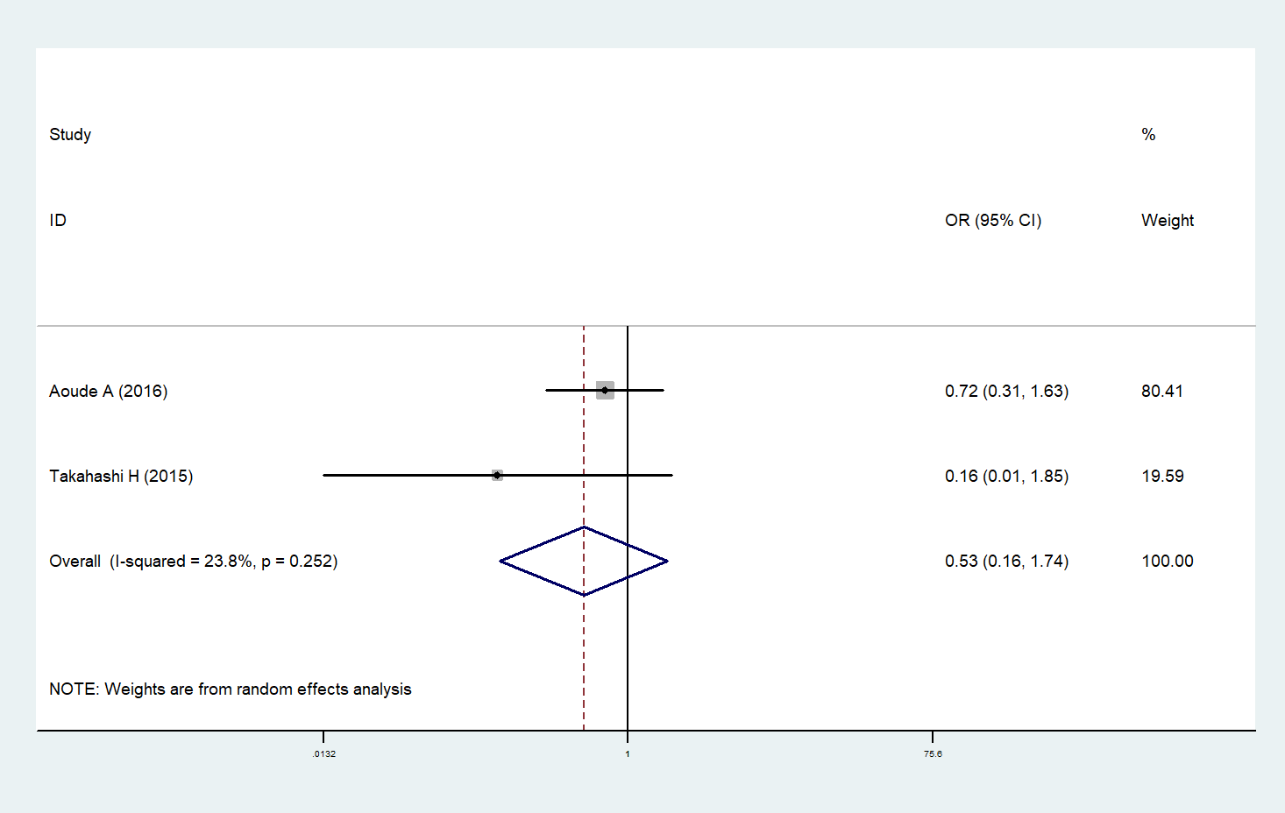


Supplemental Figure 8 Forest plot showing relationship between surgical site and incidence of VTE after spine surgery.
